# Sexual dimorphism of sulcal morphology of the ferret cerebrum revealed by MRI-based sulcal surface morphometry

**DOI:** 10.3389/fnana.2015.00055

**Published:** 2015-05-06

**Authors:** Kazuhiko Sawada, Miwa Horiuchi-Hirose, Shigeyoshi Saito, Ichio Aoki

**Affiliations:** ^1^Department of Nutrition, Faculty of Medical and Health Sciences, Tsukuba International UniversityTsuchiura, Japan; ^2^Division of Health Sciences, Department of Medical Engineering, Osaka University Graduate School of MedicineSuita, Japan; ^3^Center for Information and Neural Networks, National Institute of Information and Communications Technology, Osaka UniversitySuita, Japan; ^4^Molecular Imaging Center, National Institute of Radiological SciencesChiba, Japan

**Keywords:** sulcus, gyrification, neurodevelopmental disorders, carnivores, sex difference

## Abstract

The present study quantitatively assessed sexual dimorphism of cortical convolution and sulcal morphology in young adult ferrets by MRI-based sulcal surface morphometry. *Ex vivo* T_1_-weighted (short TR/TE) MRI of the ferret cerebrum was acquired with high spatial resolution at 7-tesla. The degree of cortical convolution, evaluated quantitatively based on 3D MRI data by sulcation index (SI), was significantly greater in males (0.553 ± 0.036) than in females (0.502 ± 0.043) (*p* < 0.001). The rostrocaudal distribution of the cortical convolution revealed a greater convolution in the frontal region of the cortex in males than in females and by a posterior extension of the convolution in the temporo-parieto-occipital region of males. Although the cerebral width in the frontal region was not different between sexes, the rhinal fissure and rostral region of splenial sulcus were more infolded in males than in females. On the contrary, the cerebral width was greater in males in the temporo-parieto-occipital region, and male-prominent posterior extension of infolding was noted in the lateral sulcus, caudal suprasylvian sulcus, pesudosylvian sulcus, hippocampal sulcus, and the caudal region of splenial sulcus. Notably, the caudal descending region of lateral sulcus was clearly infolded in males, but obscured in females. The present results suggest a region-related sexual dimorphism of the sulcal infolding, which is reflected by local cortical expansion in the ferret cerebrum. In particular, male-favored sulcal infolding with expansion of the temporo-parieto-occipital neocortex may be relevant to the human cerebral cortex regarding visuo-spatial and emotion processing, which are known to differ between sexes. The present results will provide fundamental information assessing sex-related changes in the regional sulcal infolding, when ferrets with experimentally-induced gyrification abnormality will be used as models for male-prevalent or male-earlier-onset neurodevelopmental disorders.

## Introduction

In some mammalian species, the cerebral cortex forms a gyrencephalic morphology with phylogenetically-conserved patterns of sulci and gyri (Chi et al., 1977; Sawada et al., 2012a, 2014). While sexual dimorphism of the sulcal morphology has not been fully addressed, an asymmetric pattern of the primary sulcal length is known to differ between sexes in humans (Liu et al., 2010) and cynomolgus monkeys (Imai et al., 2011). The male adult pattern of sulcal length asymmetry in those primates was acquired during adolescence to young adulthood (Clark et al., 2010; Sakamoto et al., 2014). Also, age-related changes in sulcal morphology are more prominent in males than in females (Kochunov et al., 2005). Abnormal development of the primary sulci is reportedly involved in pathological changes in human psychological and neurodevelopmental disorders such as schizophrenia, obsessive-compulsive disorder and autism (Levitt et al., 2003; Boddaert et al., 2004; Wobrock et al., 2010; White and Hilgetag, 2011). The onset and the incidences of some of those neurodevelopmental disorders are different between sexes (Rossi et al., 1994; Kulynych et al., 1997; Vogeley et al., 2000; Levitt et al., 2003; Harden et al., 2004). However, sexual differences in the sulcal morphology and gyrification in the male-prevalent neurodevelopmental disorders have not been documented.

Use of model animals will facilitate understanding of the gyrification mechanism and pathogenesis of neurodevelopmental disorders with gyrification abnormalities. Ferrets (*Mustela putorius*) are small laboratory animals, bearing cerebral sulci (Lawes and Andrews, 1998) patterns comparable to those in other carnivores, cats (Ferrer et al., 1988; Smith et al., 2001), and dogs (Wosinski et al., 1996). This animal has advantageous characteristics as a model animal for investigating the sulcal emergence and pathogenesis of neurodevelopmental disorders related with gyrification abnormalities as follows. (1) The ferret is a prolific animal (Fox, 1998). It is easier to collect ferret offspring than offspring of non-human primates when carrying out experimental studies. (2) The developmental stages of gyrification in primates on the basis of cerebral growth and gyrification (Sawada et al., 2012b) can be applied to ferrets (Sawada, 2014), although their sulcal and gyral patterns were distinct from those in primates. (3) Ferrets experience sulcation during the first 2 weeks of postnatal age (Smart and McSherry, 1986; Sawada and Watanabe, 2012), in contrast to the sulcal emergence in primates during fetal period. This allows one to apply experimental magnifications (i.e., drug administration) directly to ferret pups during the sulcal emergence. (4) The small size of the ferret cerebrum (3.0–3.3 cm length and 0.7–0.9 cm width in the adult) (Sawada and Watanabe, 2012) allows an easier histological approach compared to primates. Recently, greater region-specific volumes of the cortex and subcortical white matter have been found in males rather than females in the ferret cerebrum (Sawada et al., 2013). In this report, we revealed a signal enhancement from *ex vivo* T_1_-weighted MRI of the male cortex (Sawada et al., 2013), which is considered to reflect increasing axonal caliber rather than myelin sheath thickness (Perrin et al., 2008). In our previous neuroanatomical study, a greater cortical convolution in males than in females was observed in the visual cortical area of ferrets following the completion of the primary sulcal emergence (Sawada and Watanabe, 2012). However, the cortical folding and sulcal infolding in the ferret cortex have not been quantitatively accessed. The present study aimed to characterize sexual dimorphism of gyrification and sulcal morphology in the cerebrum of ferrets. The current results will provide fundamental information evaluating quantitatively sex-related changes in the regional sulcal infolding, when ferrets with experimentally-induced gyrification abnormality will be used as models for male-prevalent or male-earlier-onset neurodevelopmental disorders. In order to achieve a sufficient resolution for determining individual structures, we used *ex vivo* T_1_-weighted MRI with a high spatial resolution 7-tesla MR system to evaluate sulcal morphology quantitatively.

## Materials and methods

### Samples

The present study utilized cerebra from male and female ferrets at postnatal day (PD) 90 (male, *n* = 5; female, *n* = 5). The animals were purchased from SLC (Hamamatsu, Japan). After bringing them to our laboratory, they were deeply anesthetized with an intraperitoneal injection of chloral hydrate (400 μg/g body weight), and were perfused with 0.9% NaCl followed by 4% paraformaldehyde (PFA) in a 10 mM phosphate buffer, pH 7.4. These were the same samples that had been previously used in our gross anatomical examination of sulcation in ferrets (Sawada and Watanabe, 2012).

### MRI measurements

MRI measurements were carried out as with our previous study (Sawada et al., 2013). Three-dimensional T_1_-weighted MRI (short TR/TE) was performed with a 7.0-T MRI system (Magnet; Kobelco and Jastec, Kobe, Japan) (Console; Bruker BioSpin, Ettlingen, Germany). A birdcage RF coil for transmission and reception (70 mm inner diameter, Rapid Biomedica; or 60 mm inner diameter, Bruker BioSpin) was used with a field of view adequate for the sample dimensions. Slice orientation (transaxial) was precisely adjusted for the cerebral base using pilot-MR images obtained by gradient-echo sequence. Three-dimensional T_1_-weighted images covering the entire brain were acquired using the rapid acquisition with relaxation enhancement (RARE) sequence, with the following parameters: repetition time (TR) = 300 ms, echo time (TE) = 9.6 ms (effective TE = 19.2 ms), RARE factor = 4, field of view (FOV) = 32 × 32 × 40 mm^3^, acquisition matrix = 256 × 256 × 256, voxel size = 125 × 125 × 156 μm^3^, number of acquisitions (NEX) = 2, and total scan time = 2 h 43 min 50 s.

### 3D volume-rendered images

All 3D T_1_-weighted MRI images were used. The cerebral cortex and cerebrospinal fluid areas of the primary sulci were semi-automatically segmented on MRI images using the SliceOmatic software ver 4.3, based on image contrast as well as the user's knowledge of the anatomy. Segmented images were then analyzed using the 3D-rendering module of the same software. Images of the cerebral cortex were rendered in 3D using the surface projection algorithm which best visualized the surface. Three-dimensional rendered images were then rotated and manipulated in a manner that best visualized brain morphology by a linear registration method using the software.

### Fronto-occipital (FO) length and cerebral width

**Figure 1:**
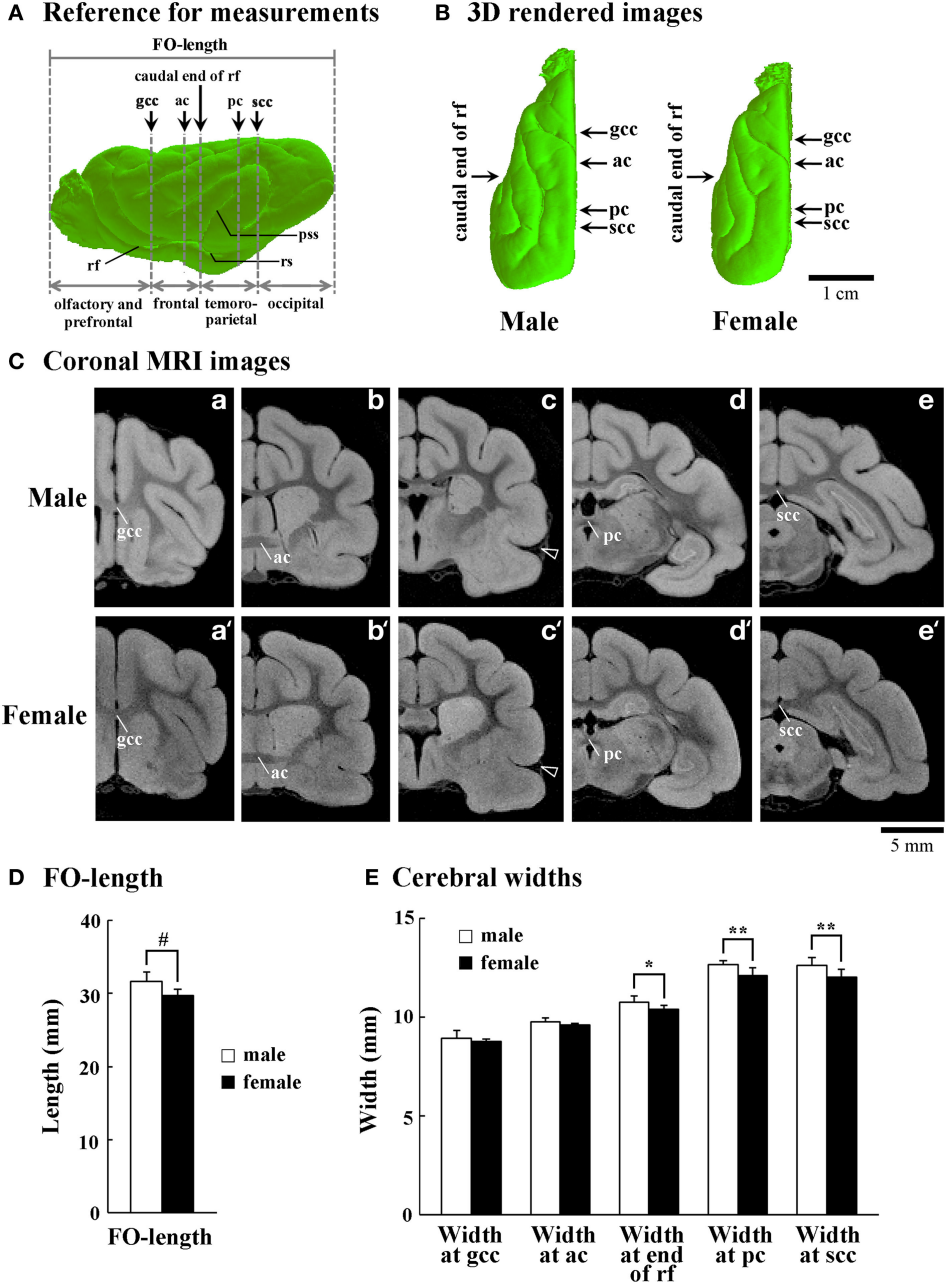
**Size and shape of cerebral hemispheres of male and female ferrets. (A)** Reference for measurements of front-occipital length (FO-length) and the width of cerebral hemispheres. Arrows indicate points for measuring cerebral width at the genu of corpus callosum (gcc), anterior commissure (ac), caudal end of rhinal fissure (rf), posterior commissure (pc), and splenium of corpus callosum (scc). Four subdivisions of the cerebral hemisphere, the boundaries of which are defined on the basis of the structural landmarks on coronal MRI images, are also indicated. **(B)** Three-dimensional rendered images of the left cerebral hemisphere of male and female ferrets (dorsal view). Arrows indicate points for measuring cerebral width at the gcc, ac, caudal end of rf, pc, and scc. **(C)** Coronal T_1_-weighted (short TR/TE) MRI for measuring the cerebral hemisphere at the gcc (a,a'), ac (b,b'), caudal end of rf (c,c'), pc (d,d'), and scc (e,e'). Arrowheads in (c) and (c') indicate the beginning of pseudosylvian sulcus (pss). **(D)** Bar graphs for FO-length of the cerebral hemisphere. ^#^*P* < 0.001 (Student's *t*-test). **(E)** Bar graphs for the width of cerebral hemisphere. ^*^P < 0.01, ^**^P < 0.001 (Scheffe's test).

The fronto-occipital (FO) length from the frontal pole to the occipital pole of the cerebral cortex was measured with the 3D-rendered images (Figure 1A) using SliceOmatic software. The width of the cerebral cortex was measured from coronal MRI images at the genu of corpus callosum, the anterior commissure, the caudal end of rhinal fissure, the posterior commissure and the splenium of corpus callosum (scc) (Figures 1B,C) using the same software.

### Cortical surface area and sulcation index

All 3D T_1_-weighted MRI images were used for measurements. According to the criteria in our previous study (Sawada et al., 2014), a clear indentation at the cerebral surface with curvature of the adjacent subcortical white matter was considered the indication of a sulcus. Digital surface analysis techniques that attempt to identify narrow sulci based on measured surface curvature will fail (Sawada et al., 2014), but small indentations of the cerebral sulci could be defined using our criteria. Furthermore, a gyrus was defined as any tissue delimited by two or more fissures, sulci, or dimples. In some cases, a rostrocaudal continuation on the cerebral surface was formed by two or three sulci: the cruciate sulcus, splenial sulcus, and retrosplenial sulcus; the coronal sulcus and lateral sulcus; and the rostral suprasylvian sulcus and caudal suprasylvian sulcus. A confluence of the ancinate sulcus made a boundary between the coronal sulcus and lateral sulcus (Lawes and Andrews, 1998). Boundaries of other sulci were defined by their discontinuous linkages obtained by coronal or sagittal MRI images (Supplemental Figure 1). The terminology and identification of cerebral sulci and gyri were based on the textbook by Lawes and Andrews (1998).

The cortical surface area excluding sulcal grooves (closed surface area) and the surface areas composed of sulcal grooves (sulcal area) were separately computed from 3D MRI using the SliceOmatic software. The sum of those areas was defined as the cortical surface area. The degree of cortical convolution was evaluated quantitatively based on 3D MRI data by sulcation index (SI) according to our previous procedure (Sawada et al., 2014), which was a modified procedure originally designed by Dubois et al. (2008). The SI was calculated by ratios between the closed surface area and the sulcal areas (Supplemental Figure 2).

### Rostrocaudal distributions of cortical contours, gyrification index, and areas of cortex

All 3D T_1_-weighted MRIs were used for measurements. The outer contour represented the most superficial region of the cortex surrounding the external gyral surfaces, excluding the inner sulci (Supplemental Figure 3). The inner contour formed the adjacent boundary of the outer contour in addition to the sulcal groove (Supplemental Figure 3). Measurements were obtained semi-automatically by tracing the outer contours of the cortex and the surfaces of all sulcal grooves on coronal MRI using the SliceOmatic software. The gyrification index (GI) of cortical convolution and sulcal infolding on each coronal MRI was estimated by ratios of the outer contour, with the sum of sulcal surfaces or the surface of each sulcus (Supplemental Figure 3). Areas of the cerebral cortex on all coronal MRIs were also segmented semi-automatically using the same software. For making rostrocaudal distribution maps of each measurement, the coronal MRI at the anterior commissure was registered as “slice number 0.” The means of each perimeter were calculated on all coronal MRI slices, and the rostrocaudal course of each perimeter's distributions was represented throughout the cerebral cortex.

In the present study, the cerebral cortex was roughly divided into four regions at a rostrocaudal axis. The regions' boundaries were defined based on the structural landmarks on coronal MRI images, which were used for measuring cerebral width (Figure 1C). The region between the frontal pole and the genu of corpus callosum (until the slice number was approximately −20) was defined as the olfacto-prefrontal region; the region between the genu of corpus callosum and the caudal end of rhinal fissure (the slice number was approximately between −20 and 10) as the frontal region; the region between the caudal end of rhinal fissure and scc (the slice number was approximately between 10 and 55) as the temporo-parietal region; and the region between the scc and the occipital pole (the slice number was approximately 55 or greater) as the occipital region (Figure 1A). By our definition, the primary motor cortex was present in the frontal region (Foxworthy and Meredith, 2011). The pseudosylvian and lateral sulci cross the temporo-parietal region, and run through the primary auditory and auditory associated cortical areas (Keniston et al., 2009) and the parietal cortex (Manger et al., 2002), respectively. The caudal region contains the visual cortical area (Manger et al., 2004; Homman-Ludiye et al., 2010).

### Statistical analysis

All measurements of the left and right hemispheres were quantified separately. This was followed by a paired sampled *t*-test that demonstrated no significant left/right differences, and data on each side were considered to be “*n* = 1.”

Significant differences in the cortical surface areas, SI of the cortex, and FO-length of the cerebral hemisphere between sexes were evaluated statistically by One-Way ANOVA, followed by a two-tailed Student's *t*-test. Sex-related changes in areas and SI of each primary sulcus, and the cerebral widths, were statistically evaluated by Two-Way ANOVA using both sexes and primary sulci as factors. Then, as *post-hoc* testing, Scheffe's test was used to compare males and females.

### Ethics

The experimental procedures in the present study were conducted in accordance with the guidelines of the National Institutes of Health (NIH) for the Care and Use of Laboratory Animals. The Institutional Animal Care and Use Committee of Tsukuba International University approved the procedures, and all efforts were made to minimize the number of animals used and their suffering.

## Results

### Surface areas of cerebral cortex and sulcal grooves

As was true with the cortical volume reported in our previous study (Sawada et al., 2013), the surface area of cerebral cortex was significantly greater in males (1332.9 ± 59.2 mm^2^) than in females (1216.7 ± 53.5 mm^2^) (*p* < 0.001) (Table 1). This sex difference involved a region-related change in the sulcal surface areas. Two-Way ANOVA revealed significant effects on both sexes [*F*_(1, 270)_ = 91.486, *p* < 0.001], the primary sulci [*F*_(14, 270)_ = 681.045, *p* < 0.001], and the interactions of the two [*F*_(14, 270)_ = 3.338, *p* < 0.001]. *Post-hoc* testing indicated significantly greater surface areas in males than in females in the presylvian sulcus (*p* < 0.01), rhinal fissure (*p* < 0.001), lateral sulcus (*p* < 0.001), caudal suprasylvian sulcus (*p* < 0.001), pseudosylvian sulcus (*p* < 0.001), splenial sulcus (*p* < 0.001), and hippocampal sulcus (*p* < 0.001).

**Table 1:** **Surface areas and sulcation index (SI) of cerebral cortex and primary sulci in male and female ferrets**.

	***n* =**	**Surface area (mm^3^)**	**SI**
**CEREBRAL CORTEX**			
Male	10	1332.9 ± 59.2^##^	0.553 ± 0.036^#^
Female	10	1216.7 ± 53.5	0.502 ± 0.043
**PRESYLVIAN SULCUS (prs)**			
Male	10	60.9 ± 7.3^**^	0.061 ± 0.006
Female	10	55.2 ± 4.4	0.059 ± 0.004
**RHINAL FISSURE (rf)**			
Male	10	91.9 ± 5.5^***^	0.109 ± 0.007^**^
Female	10	83.2 ± 5.9	0.103 ± 0.006
**CRUCINATE SULCUS (crs)**			
Male	10	14.7 ± 2.9	0.015 ± 0.003
Female	10	11.5 ± 1.8	0.012 ± 0.002
**CORONAL SULCUS (cns)**			
Male	10	35.8 ± 4.3	0.039 ± 0.006
Female	10	32.5 ± 3.2	0.037 ± 0.005
**LATERAL SULCUS (ls)**			
Male	10	32.8 ± 4.8^***^	0.028 ± 0.004^*^
Female	10	25.4 ± 6.1	0.023 ± 0.005
**ROSTRAL SUPRASYLVIAN SULCUS (rsss)**			
Male	10	37.5 ± 3.0	0.037 ± 0.003
Female	10	35.9 ± 3.8	0.037 ± 0.005
**CAUDAL SUPRASYLVIAN SULCUS (csss)**			
Male	10	35.7 ± 3.1^***^	0.029 ± 0.002^*^
Female	10	26.1 ± 2.9	0.024 ± 0.003
**PSEUDOSYLVIAN SULCUS (pss)**			
Male	10	25.4 ± 3.2^***^	0.023 ± 0.003^*^
Female	10	17.7 ± 3.9	0.018 ± 0.004
**RHINAL SULCUS (rs)**			
Male	10	13.2 ± 3.3	0.011 ± 0.003
Female	10	12.6 ± 4.7	0.012 ± 0.004
**SPLENIAL SULCUS (ss)**			
Male	10	111.8 ± 6.9^***^	0.111 ± 0.005^***^
Female	10	96.5 ± 10.8	0.102 ± 0.013
**RETROSPLENIAL SULCUS (rss)**			
Male	10	19.7 ± 2.9	0.020 ± 0.003
Female	10	18.5 ± 4.2	0.020 ± 0.003
**OLFACTORY SULCUS (olfs)**			
Male	10	17.0 ± 1.2	0.019 ± 0.001
Female	10	12.8 ± 1.0	0.016 ± 0.001
**HIPPOCAMPAL SULCUS (his)**			
Male	10	26.5 ± 5.7^***^	0.021 ± 0.004^*^
Female	10	19.0 ± 6.3	0.016 ± 0.004
**OCCIPITOTEMPORAL SULCUS (ots)**			
Male	10	4.8 ± 2.2	0.007 ± 0.003
Female	10	3.0 ± 1.5	0.004 ± 0.002
**OTHER SULCI**			
Male	10	16.6 ± 5.6	0.021 ± 0.008
Female	10	13.3 ± 2.6	0.019 ± 0.003

*SI of the cerebral cortex indicates the mean SI throughout the cerebral cortex*.

# *P < 0.05*,

## *P < 0.001 vs. females (Student's t-test)*.

* *P < 0.05*,

** *P < 0.01*,

*** *P < 0.001 vs. females (Scheffe's test)*.

### SI

Surface areas of the sulcal grooves revealed the absolute size of each sulcus. Next, we attempted to clarify the involvement of each sulcal infolding with the degree of cortical convolution. The SI was calculated based on 3D MRI. When the SI of the ferret cerebral cortex was compared between sexes, a highly convoluted cortical surface was noted in males (0.553 ± 0.036) rather than in females (0.502 ± 0.043) (*p* < 0.05) (Table 1). The SI of representative primary sulci was also estimated. Two-Way ANOVA revealed significant effects on both sexes [*F*_(1, 270)_ = 31.652, *p* < 0.001] and sulci [*F*_(14, 270)_ = 753.518, *p* < 0.001], but not on the interactions of the two. *Post-hoc* testing indicated very significant infolding of the rhinal fissure (*p* < 0.01), lateral sulcus (*p* < 0.05), caudal suprasylvian sulcus (*p* < 0.05), pesudosylvian sulcus (*p* < 0.05), splenial sulcus (*p* < 0.001), and hippocampal sulcus (*P* < 0.05).

### Size and shape of cerebral hemisphere

The FO-length and width of cerebral hemispheres were examined to determine whether or not sex-related changes in the size and shape of cerebral hemispheres were involved in sexual dimorphism of the sulcal infolding. Three-dimensional rendered images revealed that male ferrets have a relatively trigonal-shaped cerebrum larger than the female cerebrum (Figure 1B). Moreover, the FO-length was significantly greater in males than in females (*p* < 0.05) (Figure 1D). In contrast, a region-related sex difference in cerebral width was revealed by Two-Way ANOVA. There were significant effects on both sexes [*F*_(1, 90)_ = 45.371, *p* < 0.001] and cerebral regions [*F*_(4, 90)_ = 655.927, *p* < 0.001], and the interactions of the two [*F*_(4, 90)_ = 2.652, *p* < 0.05]. *Post-hoc* testing indicated three posterior points where cerebral width was significantly greater in males: at the end of rhinal fissure (*p* < 0.01), posterior commissure (*p* < 0.001) and scc (*p* < 0.001), but there was no sex difference at two anterior points (at the genu of corpus callosum and anterior commissure) (Figure 1E). Since the caudal end of the rhinal fissure was defined as the boundary between the frontal and temporo-parietal regions in the present study (Figure 1A), male-prominent lateral expansion of the cerebrum was observed in the temporo-parietal and occipital regions, but not in the olfacto-prefrontal and frontal regions. Thus, sex-related regional difference in the lateral expansion of the cerebrum was involved in the characteristic trigonal-shaped morphology of the cerebrum of male ferrets.

### Rostrocaudal patterns of GI, cortical perimeters, and areas of cerebral cortex

**Figure 2:**
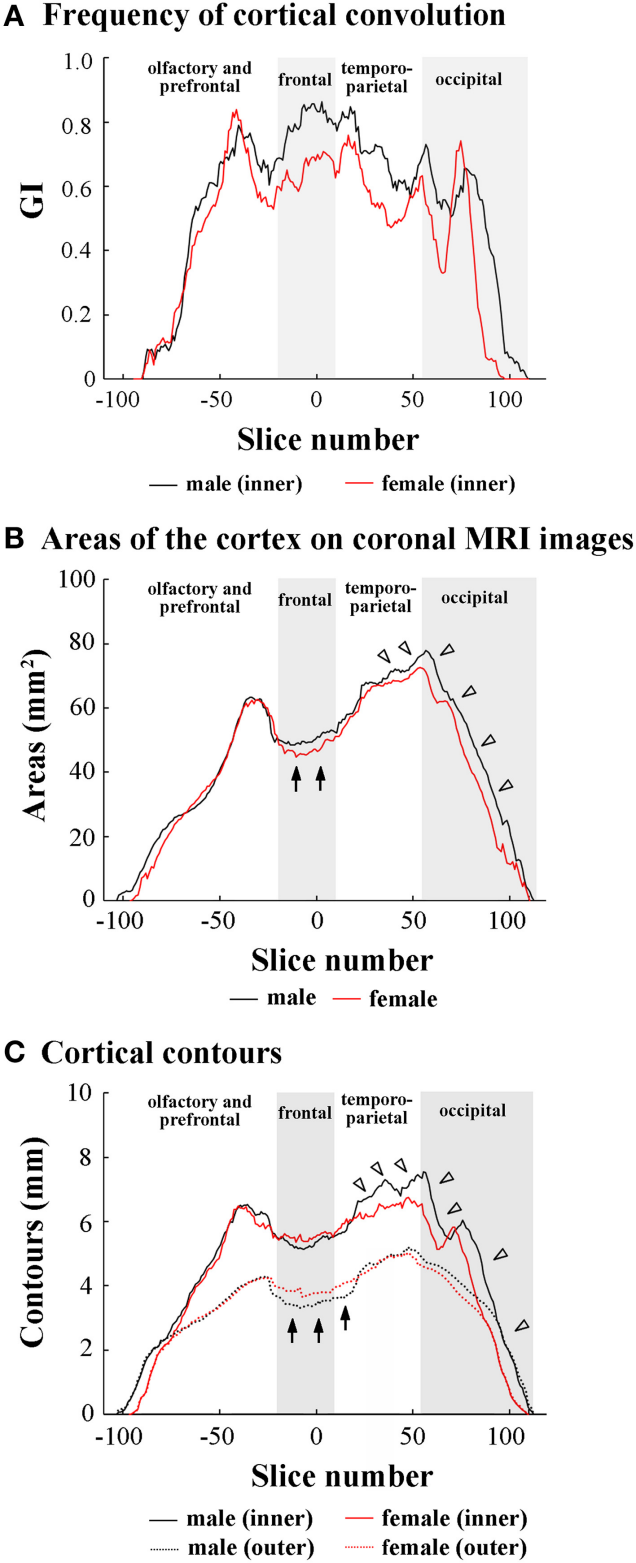
**Rostrocaudal distribution patterns of cortical convolution, area of cerebral cortex, and cortical contours. (A)** Rostrocaudal pattern of the gyrification index (GI). **(B)** Rostrocaudal pattern of the areas of cerebral cortex on coronal T_1_-weighted (short TR/TE) MRI. Arrows indicate male-over-female areas of the cortex in the frontal region. Arrowheads indicate male-favored posterior extension of areas of cortex in temporo-parietal and occipital regions. **(C)** Rostrocaudal pattern of inner and outer contours of cerebral cortex. The outer contour represented the most superficial region of the cortex surrounding the external gyral surfaces while excluding the inner sulci (Supplemental Figure 3). Arrows indicate female-over-male outer contours through the frontal to anterior 1/4 of temporo-parietal regions. Arrowheads indicate male-prominent posterior extension of inner contours. In rostrocaudal distribution maps of each measurement, the coronal MRI slice at the anterior commissure (ac) is registered as “slice number 0.” The means of each perimeter are calculated on all coronal MRI slices, and the rostrocaudal course of the distributions of each perimeter is represented throughout the cerebral cortex. Four subdivisions of the cerebral hemisphere, the boundaries of which are defined on the basis of the structural landmarks on coronal MRI images, are indicated by shadows.

Since male-over-female lateral expansion of the cerebrum was revealed in the temporo-parietal and occipital regions, but not in the olfacto-prefrontal and frontal regions of the ferret cerebrum, the region-related sexual difference in the cortical convolution and expansion along the FO axis was examined using 2D coronal MRI. Rostrocaudal GI patterns revealed a greater frequency of cortical convolution in males than in females throughout the frontal to occipital regions of the cerebrum, while the frequency was relatively low in the temporo-parietal and occipital regions (Figure 2A). However, rostrocaudal maps of the inner contour, outer contour, and entire area of the cerebral cortex showed different patterns to those found in the cortical convolution.

In the olfacto-prefrontal region, no measurements showed obvious sex differences (Figures 2A–C). In the frontal region, a greater cortical convolution was involved in male-prominent areas of the cortex on each coronal MRI (arrows in Figure 2B). However, the increased convolution of the male cortex was not accompanied by cortical expansion, because the inner contour was male-prominent (Figure 2C), and the outer contour was female-prominent (arrows in Figure 2C).

The anterior 1/4 of the temporo-parietal region had the same patterns as the frontal region regarding cortical convolution, areas of the cortex, and the inner and outer contours of the cortex. A gradual increase in areas of the cortex, but a decrease in the frequency of the cortical convolution, was shown in the posterior 3/4 of the temporo-parietal region (Figures 2A,B). Male-over-female cortical convolution in this subregion was attributed to the male-favored increment of the inner contour (closed arrows in Figure 2C), but there was no sexual difference in the outer contour (Figure 2C). On the other hand, a gradual decrease in the frequency of cortical convolution in the posterior 3/4 of the temporo-parietal region revealed that cortical expansion rather than sulcal infolding was responsible for increased areas of the cortex found with coronal MRIs in both sexes.

In the occipital region, cortical convolutions were extended posteriorly more frequently in males than in females (Figure 2A), and were involved in male-favored posterior extensions of areas of the cortex, and in the inner and outer contour of the cortex (Figures 2B,C).

### Rostrocaudal patterns of sulcal infolding

**Figure 3:**
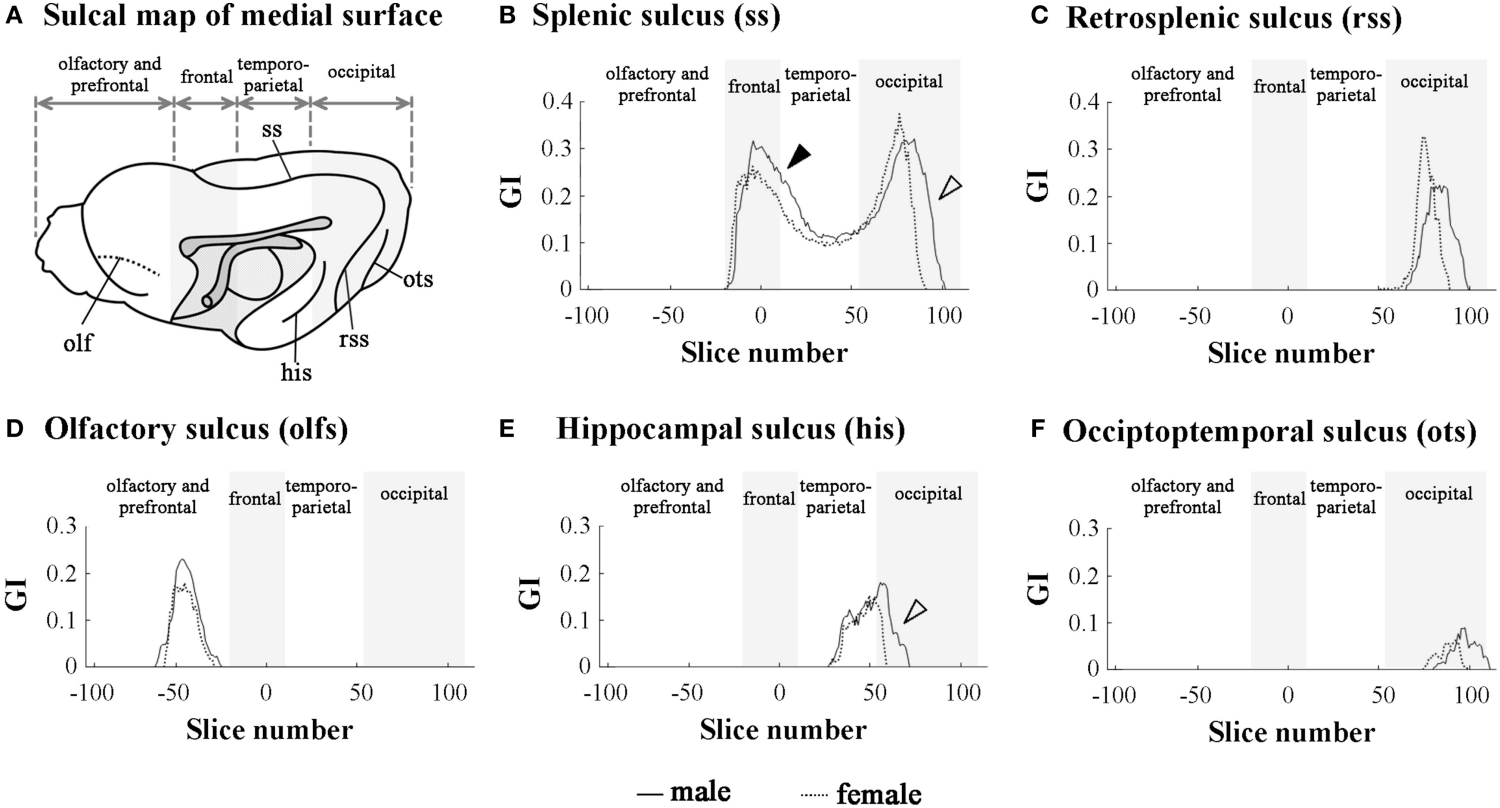
**Rostrocaudal distribution patterns of primary sulcal infolding on the medial surface of cerebral hemisphere. (A)** Sulcal map of the medial cerebral surface. Four subdivisions of the cerebral hemisphere, the boundaries of which are defined on the basis of structural landmarks on coronal T_1_-weighted (short TR/TE) MRI, are indicated by shadows. **(B)** Rostrocaudal pattern of the gyrification index (GI) of splenic sulcus (ss). Closed arrowhead indicates the ss region, which was more infolded in males than in females in the frontal region (a rostral peak of ss infolding). Open arrowhead indicates the ss region, which is extended more posteriorly in males than in females in the occipital region (a caudal peak of ss infolding). **(C)** Rostrocaudal pattern of the GI of retrosplenic sulcus infolding (rss).**(D)** Rostrocaudal pattern of the GI of olfactory sulcus infolding (olfs). **(E)** Rostrocaudal pattern of the GI of infolding of hippocampal sulcus (his). Open arrowhead indicates his region, which is extended more posteriorly in males than in females. **(F)** Rostrocaudal pattern of the GI of occipitotemporal sulcus infolding (ots). On the rostrocaudal distribution maps of each measurement, the coronal MRI slice at the anterior commissure (ac) is registered as “slice number 0.” The means of each perimeter are calculated for all coronal MRI slices, and the rostrocaudal course of the distributions of each perimeter is represented throughout the cerebral cortex. Four subdivisions of the cerebral hemisphere, the boundaries of which are defined on the basis of the structural landmarks on coronal MRI, are indicated by shadows.

**Figure 4:**
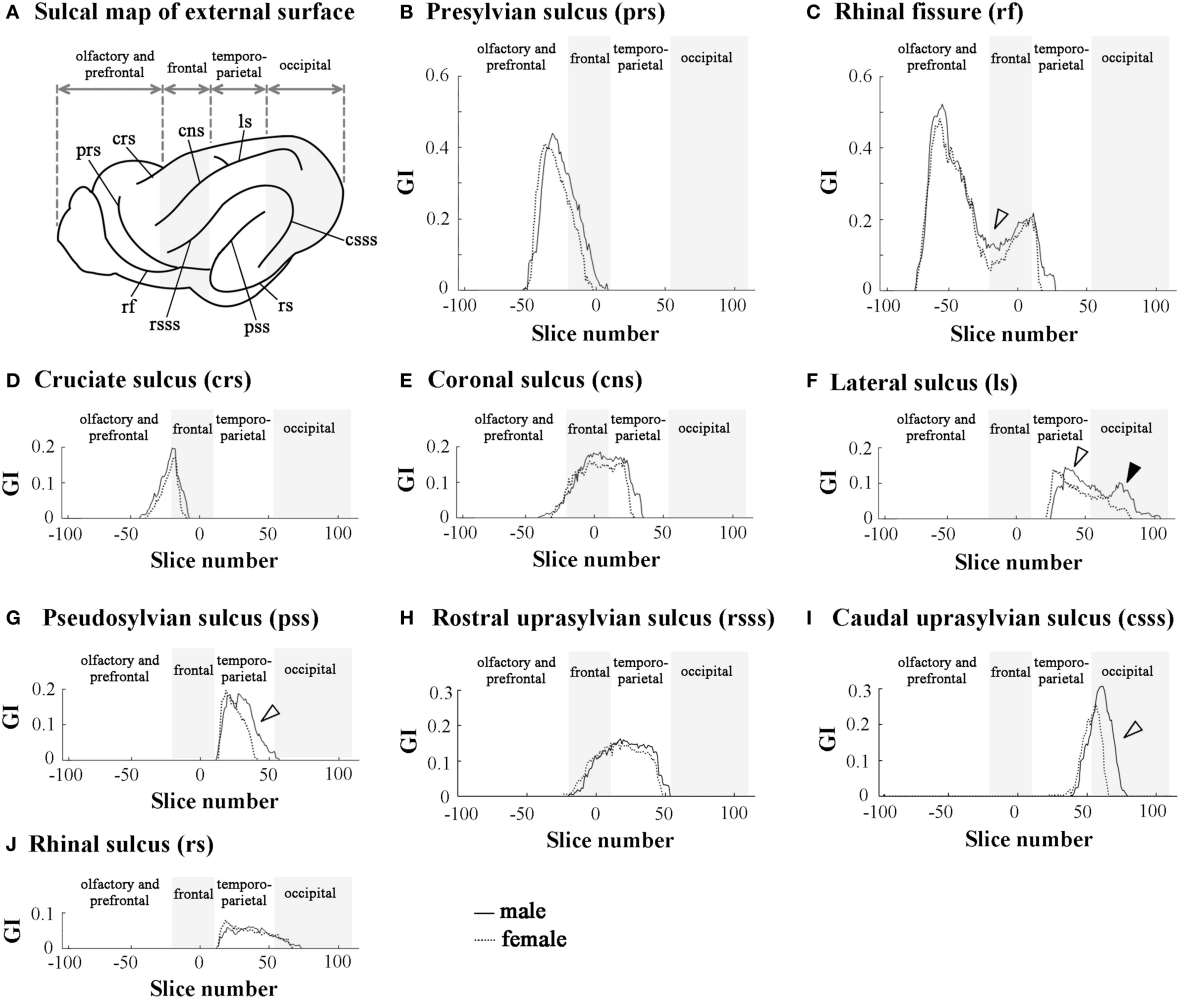
**Rostrocaudal distribution patterns of primary sulcal infolding on external surface of cerebral hemisphere. (A)** Sulcal map of the external cerebral surface. **(B)** Rostrocaudal pattern of the GI of presylvian sulcus infolding (prs). **(C)** Rostrocaudal pattern of the GI of rhinal fissure infolding (rf). Open arrowhead indicates the rf region, which is more infolded in males than in females. **(D)** Rostrocaudal pattern of the GI of cruciate sulcus infolding (crs). **(E)** Rostrocaudal pattern of the GI of coronal sulcus infolding (cns). **(F)** Rostrocaudal pattern of the GI of lateral sulcus of infolding (ls). Open arrowhead indicates the ls region, which is extended more posteriorly in males than in females in temporo-parietal region, and closed arrowhead indicates the second peak of the ls infolding, which is obvious in males but obscure in females in the occipital region. **(G)** Rostrocaudal pattern of the GI of pseudosylvian sulcus infolding (pss). Open arrowhead indicates the pss region, which is extended more posteriorly in males than in females in the temporo-parietal region. **(H)** Rostrocaudal pattern of the GI of rostral suprasylvian sulcus infolding (rsss). **(I)** Rostrocaudal pattern of the GI of caudal suprasylvian sulcus infolding (csss). Open arrowhead indicates the csss region, which is extended more posteriorly in males than in females in the occipital region. **(J)** Rostrocaudal pattern of the GI of rhinal sulcus infolding (rs). On the rostrocaudal distribution maps of each measurement, the coronal MRI slice at the anterior commissure (ac) is registered as “slice number 0.” The means of each perimeter are calculated on all coronal MRI slices, and the rostrocaudal course of the distributions of each perimeter is represented throughout the cerebral cortex. Four subdivisions of the cerebral hemisphere, the boundaries of which are defined on the basis of the structural landmarks on coronal MRI images, are indicated by shadows.

In order to clarify the involvement of each sulcal infolding in the overall pattern of cortical convolution, we examined rostrocaudal infolding patterns of representative primary sulci in the cerebral cortex of male and female ferrets. The results of sulci on the medial and lateral cerebral surfaces were shown in Figures 3, 4. In the olfacto-prefrontal region, the olfactory sulcus was infolded. The presylvian and cruciate sulci extended through the olfacto-prefrontal region to the frontal region of the external surface, and the rostrocaudal GI patterns of those three sulci were not sexually different (Figures 3D, 4B–D). The rhinal fissure extends through the olfacto-prefrontal to frontal regions, and borders on the neocortex (orbital gyrus) with the olfactory bulb or allocortex (piriform cortex) on the ventral side of the external surface. There were two peaks of rhinal fissure infolding in the olfacto-prefrontal and frontal regions, respectively. The rostral peak in the olfacto-prefrontal region demarcated the orbital gyrus and olfactory bulb, and the caudal peak in the frontal region delineated the orbital gyrus with the piriform cortex. Sex difference of the rhinal fissure infolding was detected by the greater infolding between those two peaks in the frontal region of males rather than in females (open arrowhead in Figure 4C).

The splenial sulcus, which demarcates the neocortex and allocortex on the medial cerebral surface, extends through the frontal to occipital regions. Two peaks were revealed by the rostrocaudal pattern of the splenial sulcus infolding: the rostral one in the frontal region (closed arrowhead in Figure 3B), and the caudal one, corresponding to the descending part of splenial sulcus in the occipital region (open arrowhead in Figure 3B). Sex difference in the splenial sulcus infolding was noted by a greater rostral peak in males than in females, and by a male-favored posterior extension of the caudal peak (Figure 3B). Through the frontal to temporo-parietal regions, the coronal and rostral suprasylvian sulci were transversally infolded on the external surface. Rostrocaudal GI patterns of those two sulci were not sexually different (Figures 4E, H). Thus, male-prominent cortical convolution in the frontal region as shown in Figure 2B was attributed to male-over-female infolding of the rhinal fissure and splenial sulcus.

Through the temporo-parietal to occipital region, the pseudosylvian sulcus was infolded in the temporo-parietal region, and the lateral and caudal suprasylvian sulci extended transversally on the external surface of the neocortex. As sulci demarcating the allocortex, hippocampal and rhinal sulci were infolded. In the temporo-parietal region, male-prominent infolding with posterior extension was noted in lateral and pseudosylvian sulci (open arrowheads in Figures 4F, G). Notably, the lateral sulcus clearly had a second peak of infolding in the occipital region of males, but it was obscured in females (closed arrowheads in Figure 4F). In contrast, the hippocampal and caudal suprasylvian sulci were extended posteriorly in males more than in females in the occipital region (Figures 3E, 4I), as was the descending part of the splenial sulcus on the medial surface (Figure 3B). The rhinal sulcus extended on the ventral side of the external surface as a caudal continuation of the rhinal fissure. The rostrocaudal pattern of rhinal sulcus infolding was, however, not sexually different in the temporo-parietal region, or even in the occipital region (Figure 4J). Within the occipital region, retrosplenial and occipitotemporal sulci were infolded on the medial surface. Although the SI of those two sulci was not sexually different (Table 1), a posterior extension of the infolding of retrosplenial and occipitotemporal sulci was observed slightly in males (Figures 3C,F). Thus, male-prominent cortical convolution in the temporo-parietal region as shown in Figure 2B was involved in greater infolding of the lateral and pseudosylvian sulci. Male-favored posterior expansion of the cortex in the occipital region as shown in Figures 2B,C was accompanied by male-over-female infolding with posterior expansion of the primary sulci.

### Sulcal morphology

**Figure 5:**
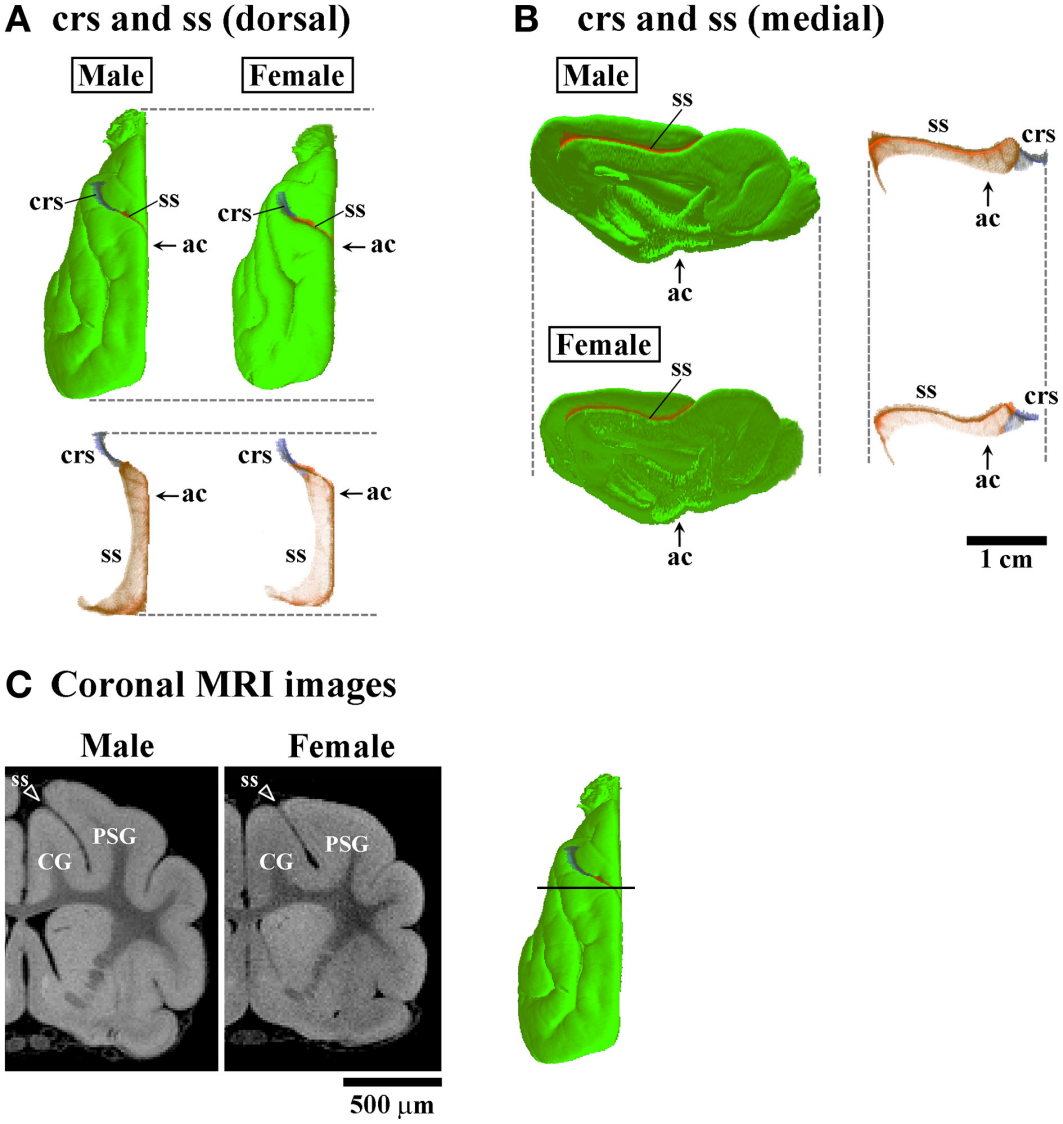
**Three-dimensional rendered images of crucinate and splenic sulci. (A)** Dorsal views of 3D-rendered images of the crucinate sulcus (crs) and splenic sulcus (ss) with or without the cerebral surface. Each image is registered at the anterior commissure (ac). **(B)** Medial views of 3D-rendered images of the crs and ss with or without the cerebral surface. Each image is registered at the ac. **(C)** Coronal T_1_-weighted (short TR/TE) MRI of the ferret cerebrum corresponding to the rostral peak of ss infolding in the frontal region (Figure 3B). Bar in 3D-rendered images of the cerebrum indicates the positions of coronal MRI images acquired. The ss demarcates the cingulate gyrus (CG) and posterior sigmoid gyrus (PSG), male-prominent ss infolding result in a greater convolution of the PSG in males than in females.

**Figure 6:**
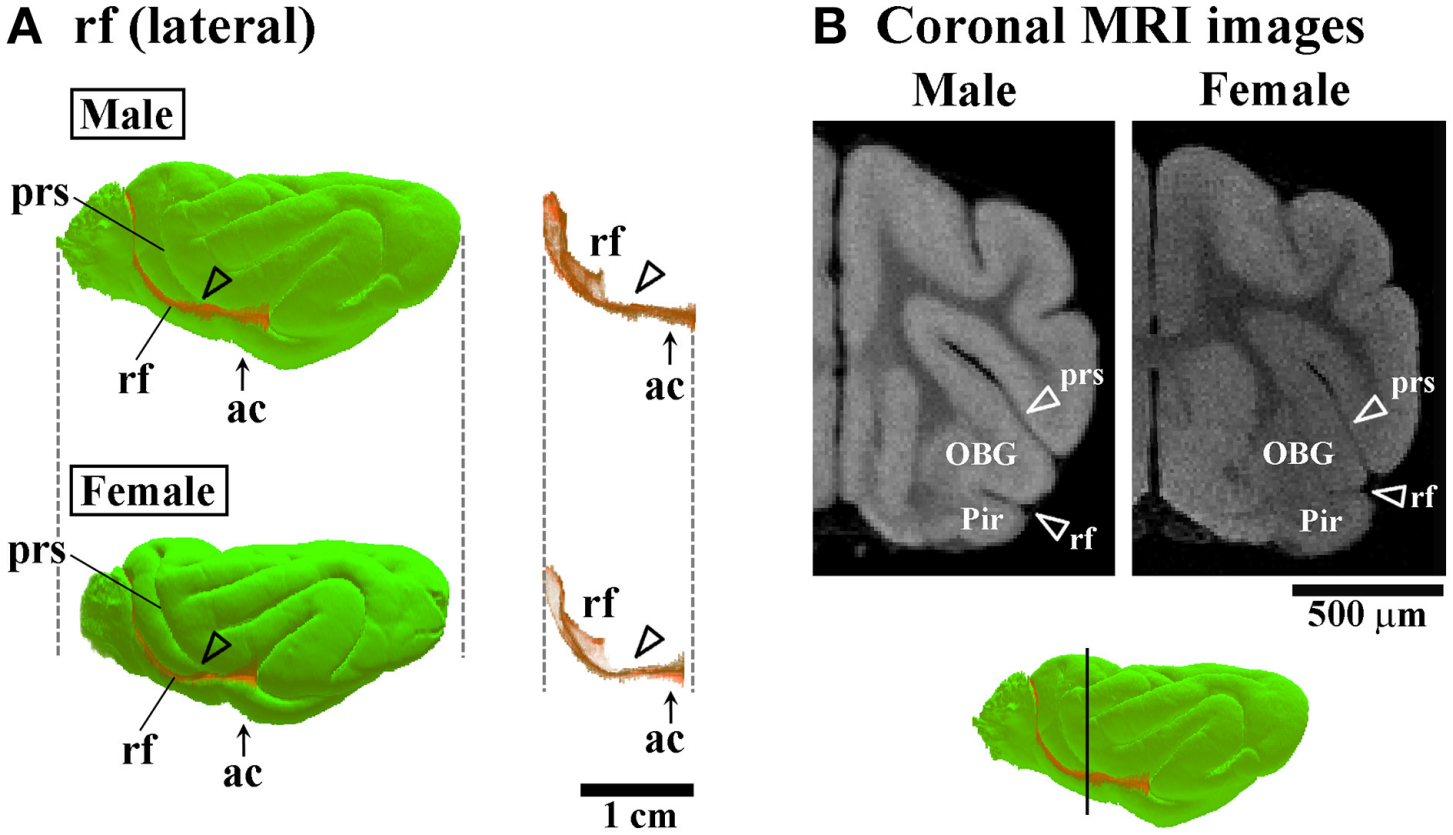
**Three-dimensional rendered images of rhinal fissure. (A)** Lateral views of 3D-rendered images of the rhinal fissure (rf) with or without the cerebral surface. Arrowheads indicate the rf region, corresponding to a second peak of the lf infolding (see open arrowheads in Figure 4C). Each image is registered at the anterior commissure (ac). **(B)** Coronal T_1_-weighted (short TR/TE) MRI of the ferret cerebrum corresponding to the rf region, which is more infolded in males than in females in the frontal region. Bar in 3D-rendered images of the cerebrum indicates the positions of coronal MRI acquired. The ss demarcates the cingulate gyrus (CG) and piriform cortex (Pir), male-prominent rf infolding result in a greater convolution of the Pir in males than in females.

**Figure 7:**
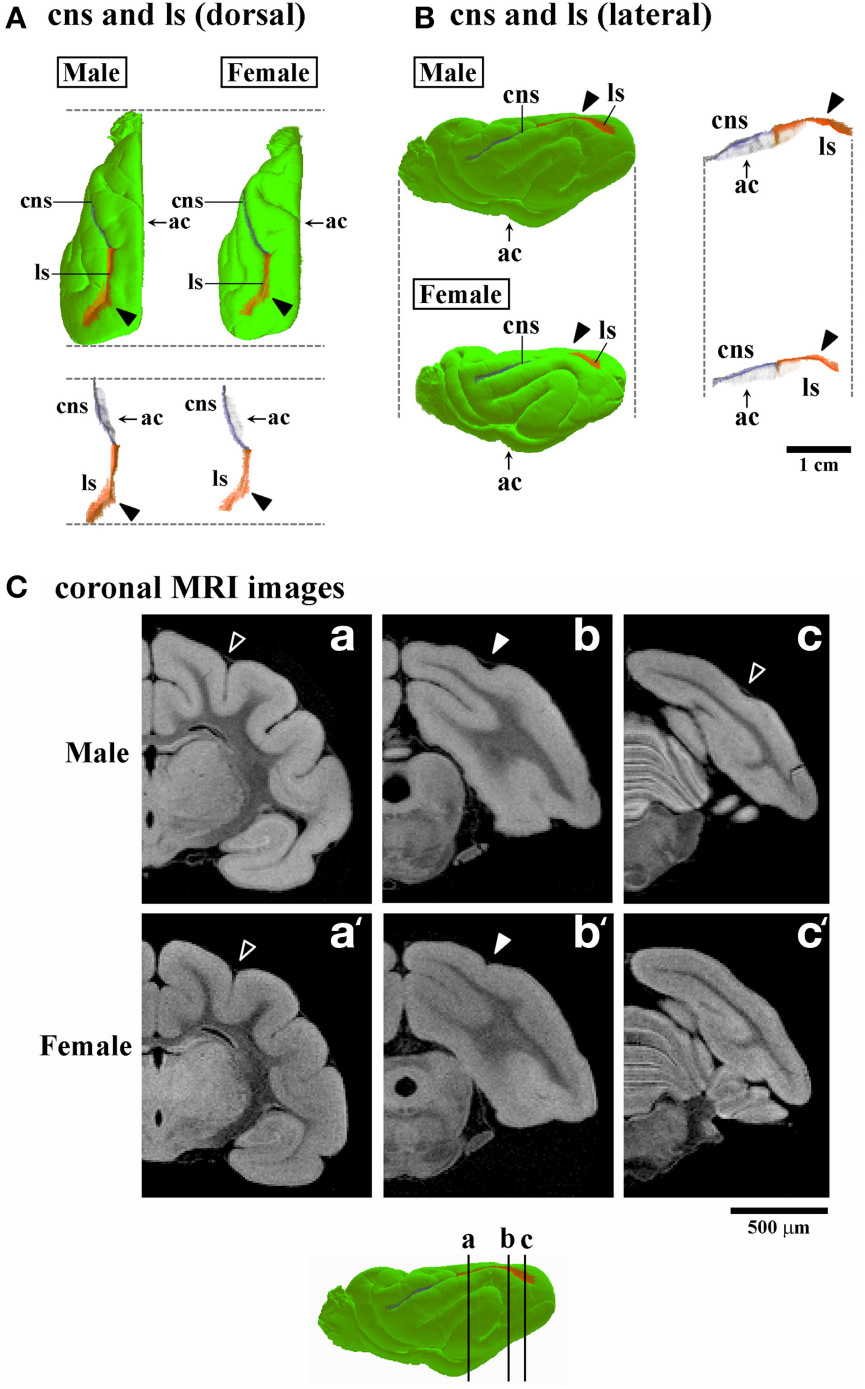
**Three-dimensional rendered images of lateral sulcus. (A)** Dorsal views of 3D-rendered images of the lateral sulcus (ls) with or without the cerebral surface. Closed arrowheads indicate the ls region corresponding to the second peak of the ls infolding, which is obvious in males, but obscure in females in the occipital region (Figure 4F). Each image is registered at the anterior commissure (ac). **(B)** Lateral views of 3D-rendered images of the lateral sulcus (ls) with or without the cerebral surface. Closed arrowheads indicate the ls region corresponding to the second peak of the ls infolding; This is obvious in males but obscure in females in the occipital region (Figure 4F). Each image is registered at the anterior commissure (ac). **(C)** Coronal T_1_-weighted (short TR/TE) MRI of the ferret cerebrum corresponding to the ls regions of the rostral peak of infolding (a,a'), the second peak of males (b) and identical region in females (b'), and near the caudal end in males (c) and identical region in females (c'). Bars in 3D-rendered images of the cerebrum indicate the positions of coronal MRI images acquired.

**Figure 8:**
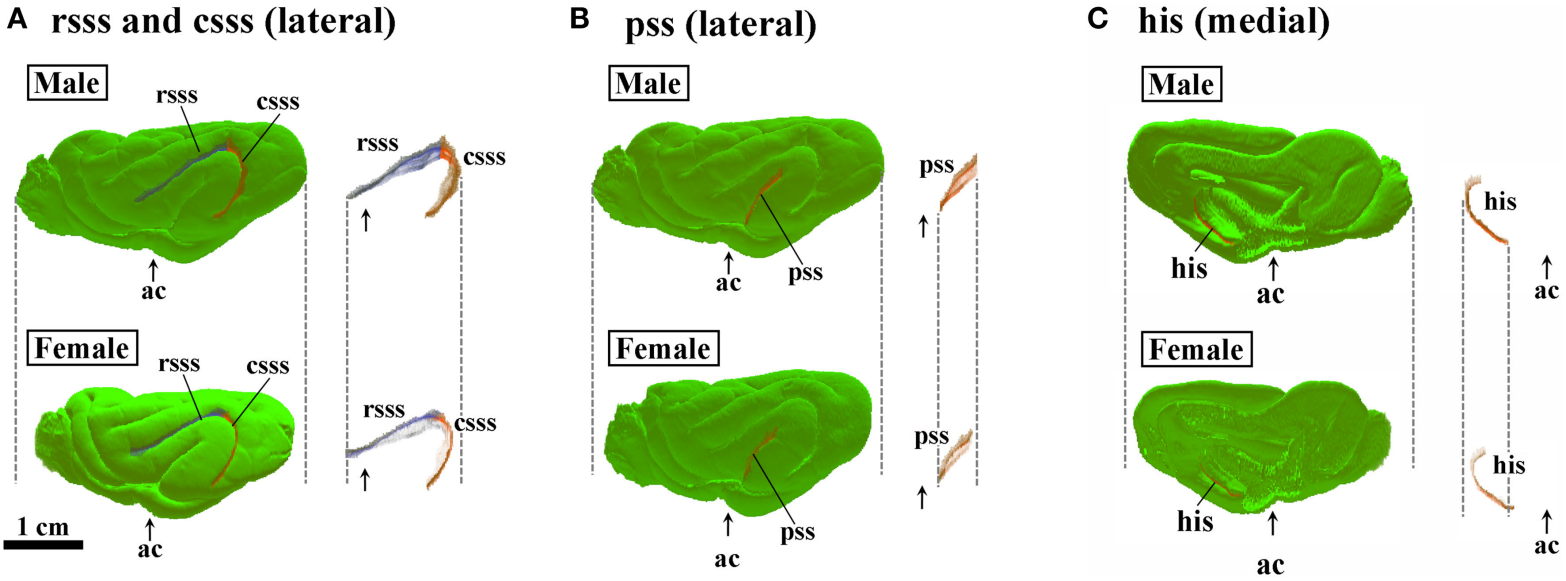
**Three-dimensional rendered images of rostral suprasylvian, caudal suprasylvian, pseudosylvian, and hippocampal sulci. (A)** Lateral views of rostral suprasylvian sulcus (rsss) and caudal suprasylvian sulcus (csss), with or without the cerebral surface. **(B)** Lateral views of pseudosylvian sulcus (pss) with or without the cerebral surface. **(C)** Medial views of hippocampal sulcus (his) with or without the cerebral surface. Each image is registered at the anterior commissure (ac).

Three-dimensional rendered images of primary sulci showing sexual dimorphic rostrocaudal infolding patterns are shown in Figures 5–8. As well as the quantitative results seen in Figures 3, 4, a male-prominent posterior extension was obtained in the splenial sulcus (Figures 5A,B), hippocampal sulcus (Figure 8C), caudal suprasylvian sulcus (Figure 8A), pseudosylvian sulcus (Figure 8B), and lateral sulcus (Figures 7A,B), when the anterior commissure of male and female cerebra was adjusted to the same point at the rostrocaudal axis. While splenial and caudal suprasylvian sulci descended in an arc on medial and external cerebral surfaces, respectively, these sulci extended posteriorly by increasing their curvatures (Figures 5A,B, 8A). Arrowheads in Figure 6A indicate the rhinal fissure infolding that was male-prominent in its rostrocaudal pattern (open arrowhead in Figure 4C). This region delineated the piriform cortex with the caudal part of orbital gyrus (Figure 6B), and made male convolution of the orbital gyrus greater than that of females on coronal MRI (Figure 6B). The lateral sulcus formed a characteristic morphology that angled and then descended laterally as a shallow groove (Figure 7A). The turning point indicated by arrowheads in Figure 7A corresponded to the caudal second peak of the lateral sulcus infolding indicated by the closed arrowhead in Figure 4F. The sex difference in the lateral sulcus morphology was obvious, particularly given by the shallower and shorter infolding found in females than in males posterior to the turning point of this sulcus (Figure 7B).

## Discussion

Sexual dimorphism and the laterality of brain structures are considered to associate with sex-related functions and functional specifications of each brain region. Some mammalian species such as primates and carnivores have a gyrencephalic morphology of the cerebral cortex (Chi et al., 1977; Smart and McSherry, 1986; Ferrer et al., 1988; Wosinski et al., 1996; Sawada et al., 2012a, 2014), which involves species-related morphological and functional specifications of the cerebrum. Recently, we have proposed the developmental stages of gyrification in primates on the basis of cerebral growth and gyrification: Stage 1. Appearance of the primary sulci demarcating cerebral lobes and limbic cortex; Stage 2. Appearance of the primary sulci demarcating neocortical gyri; Stage 3. Appearances of secondary and tertiary sulci; and Stage 4. Growth of sulcal length and depth (Sawada et al., 2012b). Such gyrification stages can be applied to a non-primate mammal, the ferret (Sawada, 2014). In the present study, sexual dimorphism of the cortical convolution in young adult ferrets was characterized by a male-preferred sulcal infolding in the frontal region and by a male-preferred posterior extension of sulcal infolding with cortical expansion in the temporo-parieto-occipital region. In our previous neuroanatomical study, a greater cortical convolution in males than in females was observed in the visual cortical area of ferrets following the completion of the primary sulcal emergence (Sawada and Watanabe, 2012). A regional pattern of sulcal length asymmetry was acquired in human males from adolescence to young adulthood (Blanton et al., 2001; Clark et al., 2010), as well as in cynomolgus monkeys (Sakamoto et al., 2014), and enhancement of sulcal length asymmetry in prefrontal and perisylvian regions during adolescence was more prominent in males than in females in humans (Blanton et al., 2001; Clark et al., 2010). Thus, sex-related change in the cortical convolution may occur at Stage 4 of the gyrification process (during adolescent to young adult periods), although its characteristics will vary depending on the species.

### Methodological issues

The present MRI-based morphometric analysis of sulcal surface characterized sexual dimorphism of the sulcal morphology in the ferret cerebrum. Removal of the skull before MRI measurements may be involved in slight artifacts in brain samples (Ma et al., 2008), but it is known that volumes of the brain do not differ between *in vivo* and *ex vivo* MRI measurements (Oguz et al., 2013). An improvement of image quality in *ex vivo* MRI measurements was considered to delineate detailed morphology of the sulci in the present study.

Two approaches exist for assessing the degree of gyrification: quantitatively, with the GI based on coronal 2D MRI data (Zilles et al., 1988); and with the SI based on 3D data (Dubois et al., 2008). The present study used the SI for evaluating overall differences in cortical convolution and infolding of the representative primary sulci. Furthermore, it examined rostrocaudal patterns of the GI of the representative primary sulci. For making rostrocaudal distribution maps, the coronal MRI at the anterior commissure was registered as “slice number 0.” Such spatial alignment allowed for a comparison of the spatial distribution of the sulcal infolding between sexes. Studying four regions of the cerebral cortex, roughly divided based on the structural landmarks on coronal MRI images (see Figure 1A), brought about further speculation regarding local changes of the sulcal infolding in particular cortical regions. Thus, sex-related changes in regional development of the ferret cerebral cortex were revealed by the present MRI-based morphometrical approaches. In combination with the T_1_-weighted MRI-based maximum intensity projection (MIP) map of the cerebrum that visualizes functional cortical areas associated with myeloarchitecture (Sawada et al., 2013), the present approach may well be useful for investigating normal and abnormal development of the functional organization of the cerebrum using conventional MRI techniques.

### Sulcal infolding in temporo-parieto-occipital region

The present study revealed that male-favored posterior extension of primary sulci in the temporo-parieto-occipital region (i.e., the hippocampal sulcus, lateral sulcus, caudal suprasylvian sulcus, pseudosylvian sulcus, and the caudal descending part of the splenial sulcus) was involved in an expansion of the cortical region in the ferret cerebrum. Regarding the hippocampal sulcus, sex difference in sulcal infolding may be associated with a 11.7% larger volume of the hippocampus in male ferrets than in female ferrets (Sawada et al., 2013). Consistently, the male's hippocampus has been reported to have a larger volume in humans (Giedd et al., 1997; Suzuki et al., 2005; Carne et al., 2006) and mice (Schlaepfer et al., 1995; Spring et al., 2007). This may be related to sexual dimorphism of hippocampal functions such as male-preferred spatial tasks (Williams et al., 1990; Gron et al., 2000; Butler et al., 2006; van Gerven et al., 2012).

The lateral, caudal suprasylvian and pseudosylvian sulci extend through the neocortex of the temporo-parieto-occipital region, and delineate borders of the lateral gyrus, suprasylvian gyrus, and/or posterior ectosylvian gyrus. Those gyri include higher-order visual cortical areas such as the posterior parietal caudal cortex, posterior parietal rostral cortex, and anterior ectosylvian visual area (Manger et al., 2002, 2004, 2005). In humans, visuospatial association areas of the parietal lobes are more expanded in men than in women (Brun et al., 2009), and a sex difference in visuo-spatial processing is noted when male or female approaches a 3D-mental rotation task: there is a male-prominent activation of the superior parietal lobule; but a female-prominent activation of the inferior fontal gyrus (Thomsen et al., 2000; Hugdahl et al., 2006). Visuo-spatial processing has also been proven to occur in carnivores (Schweid et al., 2008) and macaques (Burmann et al., 2005; Köhler et al., 2005). Moreover, a cytoarchitectural study revealed that the suprasylvian gyrus in ferrets is identical to the inferior parietal lobule in macaques (Manger et al., 2002), which is homologous with the inferior parietal lobule in humans (Watson et al., 1994). Therefore, male-favored sulcal infolding with expansion of the temporo-parieto-occipital neocortex predicts the presence of a sex difference in functions of higher-order visual cortical areas such as visuo-spatial processing.

An intriguing result of the present study is that the lateral sulcus showed a strikingly different infolding pattern between male and female ferrets. The lateral sulcus angled toward the occipital region and then descended laterally through the visual cortical area (across the cortical area 19) (Manger et al., 2005). This descending region was distinguishable as a second peak of lateral sulcus infolding on its rostrocaudal infolding map in males, but not in females. The visual cortical area was consistently more convoluted in male ferrets than in female ferrets in our previous neuroanatomical study (Sawada and Watanabe, 2012). In the occipital region, the splenial sulcus was curved descendingly on the medial surface while demarcating the visual cortical area and the allocortex (Lawes and Andrews, 1998), and extended more posteriorly in males by increasing its curvature. Thus, sex-related specifications of functions and morphology of the ferret cerebrum may be characterized by a more detailed development of the visual cortical area in males than in females.

### Sulcal infolding in frontal region

Sex difference in sulcal infolding was also observed in the frontal region of the ferret cerebrum in the present study. Male-preferred infolding of the rhinal fissure and rostral region of the splenial sulcus was involved in greater convolutions of adjacent neocortical gyri such as the cingulate and orbital gyri. In contrast, larger volumes of cingulate and fronto-orbital cortices in women rather than men were reportedly related to emotion processing (Gur et al., 2002; Mann et al., 2011; Rando et al., 2013). Such different patterns of sex-related volume changes in anatomically-identical cerebral regions between ferrets and humans suggest that there is species-specification of the sex-related functions of those cerebral regions, e.g., emotion processing.

## Conclusions

In the present study, sexual dimorphism of the cortical convolution of young adult ferrets was characterized by male-favored sulcal infolding in the frontal region, and by male-prominent posterior extension of primary sulci with cortical expansion of the temporo-parieto-occipital region. In humans, sexual dimorphism of the sulcal infolding is characterized by an enhancement of sulcal length asymmetry in males rather than in females in the prefrontal and perisylvian regions, which is acquired during adolescence (Blanton et al., 2001; Clark et al., 2010). Although the cortical surface area and the degree of primary sulcal infolding were developed symmetrically in the ferret cerebrum along with volumes of the cortex and other cerebral structures (Sawada et al., 2013), sexual dimorphism of the sulcal infolding was striking in cerebral regions, which were anatomically-identical to the human cerebral cortex in terms of visuo-spatial processing (Thomsen et al., 2000; Hugdahl et al., 2006; Brun et al., 2009) and emotion processing (Gur et al., 2002; Mann et al., 2011; Rando et al., 2013). Male-earlier-onset or male-prevalent human neurodevelopmental disorders such as schizophrenia and autism involve gyrification abnormality (Rossi et al., 1994; Kulynych et al., 1997; Vogeley et al., 2000; Levitt et al., 2003; Harden et al., 2004), and are known to show sexually-dimorphic atypical processing in response to visuo-spatial (Jiménez et al., 2010; Beacher et al., 2012) and/or emotional (Mendrek, 2007; Phillips et al., 2011) stimuli. Therefore, we concluded that sexual dimorphic characteristics of the sulcal infolding of the ferret cerebrum, which are acquired at Stage 4 of the gyrification processes (during adolescent to young adult periods), provide keys to understanding the pathogenesis of human neurodevelopmental disorders with gyrification abnormality, especially those in whom pathogenesis differs by sex.

## Author contributions

All authors had full access to all the data in the study and take responsibility for the data integrity and the accuracy of the data analysis. Study concept and design: KS. Acquisition of data: KS, MH, SS, IA. Analysis and interpretation of data: KS, MH, SS. Drafting of the manuscript: KS, IA. Critical revision of the manuscript for important intellectual content: KS, MH, SS, IA. Obtained funding: KS, IA.

### Conflict of interest statement

The authors declare that the research was conducted in the absence of any commercial or financial relationships that could be construed as a potential conflict of interest.
