# Supplementary material for: Sexual dimorphism of sulcal morphology of the ferret cerebrum revealed by MRI-based sulcal surface morphometry
Source: Front Neuroanat. 2015 May 6;9:55. doi: 10.3389/fnana.2015.00055 (PMC4422084; doi:10.3389/fnana.2015.00055)
Supplement: Supplementary file 1 [file Image1.PDF]

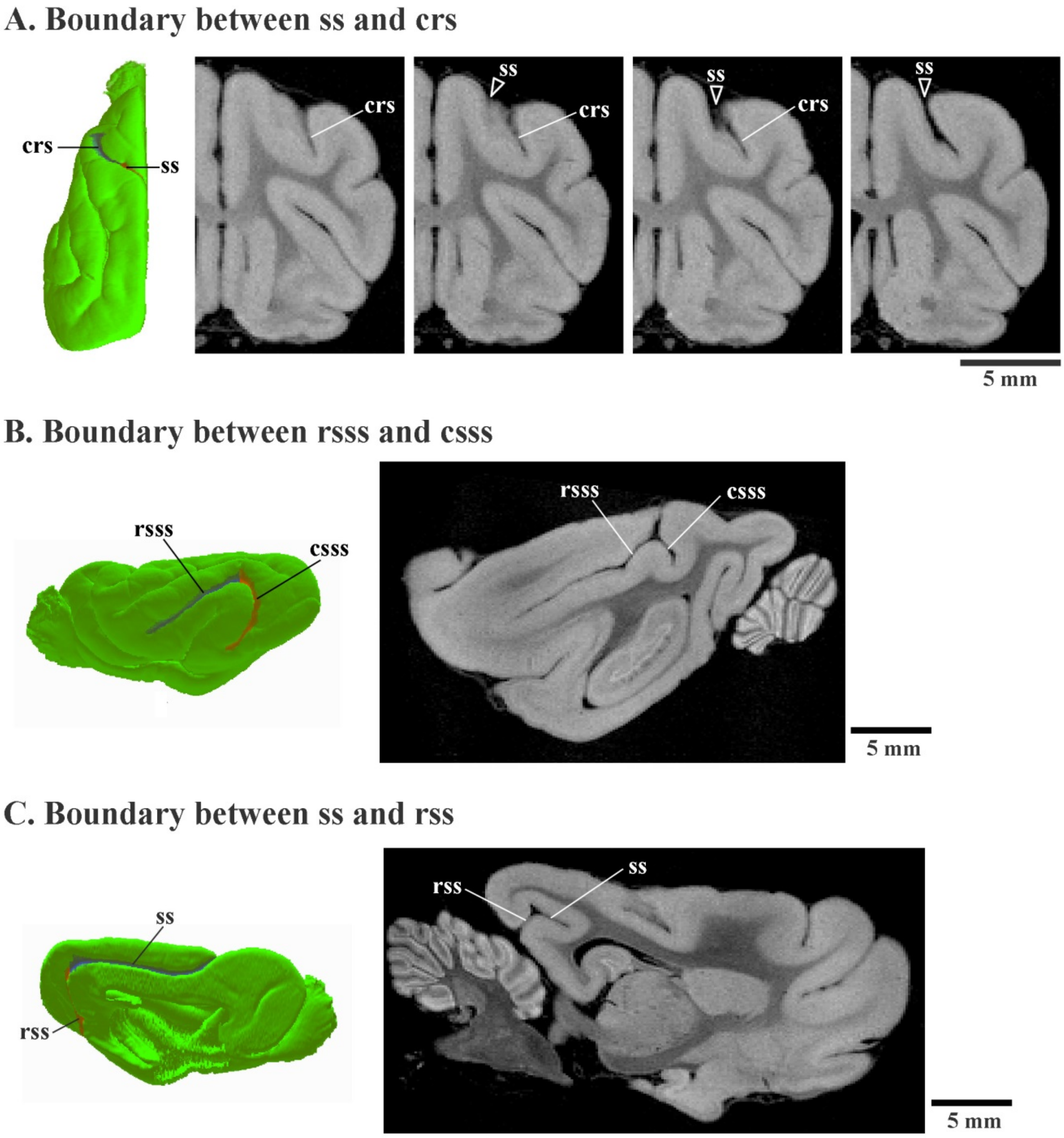

Supplemental Figure 1. Definitions of primary sulci boundaries forming a rostrocaudal continuation on the cerebral surface. (A) Boundary between the splenic sulcus (ss) and crucinate sulcus (crs). Four serial coronal T_1-weighted (short TR/TE) MRI of the frontal region of the male ferret cerebrum around boundaries between ss and crs are shown. The boundary of the ss and crs was defined by their discontinuous linkage. (B) Sagittal T_1-weighted MRI of the male ferret cerebrum indicating a boundary between the rostral suprasylvian sulcus (rsss) and caudal suprasylvian sulcus (csss). The boundary of the rsss and csss was defined by their discontinuous linkage. (C) Sagittal MRI image of the male ferret cerebrum indicating a boundary between the ss and retrosplenial sulcus (rss). The boundary between the ss and rss was defined by their discontinuous linkage.
