# Supplementary material for: Sexual dimorphism of sulcal morphology of the ferret cerebrum revealed by MRI-based sulcal surface morphometry
Source: Front Neuroanat. 2015 May 6;9:55. doi: 10.3389/fnana.2015.00055 (PMC4422084; doi:10.3389/fnana.2015.00055)
Supplement: Supplementary file 2 [file Image2.PDF]

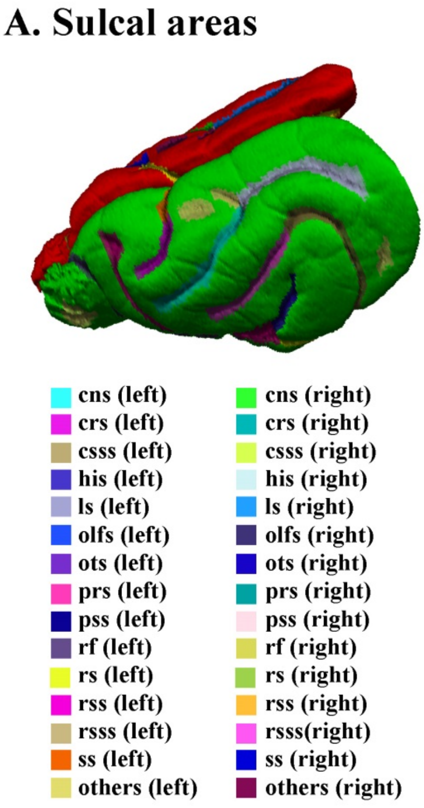

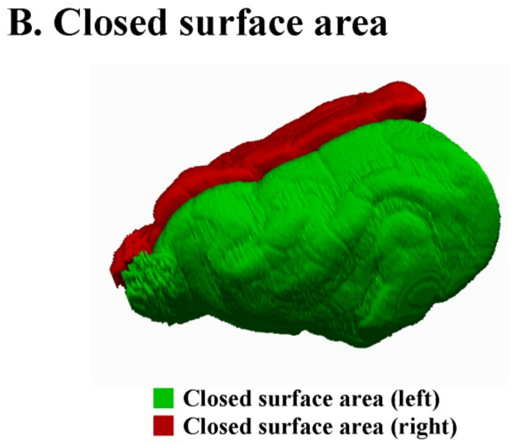

C. Formula of sulcation index (SI)

$$\text{SI (entire)} = \frac{\text{total sulcal areas}}{\text{closed surface area}}$$

$$\text{SI (sulcal)} = \frac{\text{each sulcal area}}{\text{closed surface area}}$$

Supplemental Figure 2. Procedures and formulas for calculation of sulcation index. (A) Three-dimensionally rendered images of the total surface area with segmented sulcal areas for calculating the sulcation index (SI). (B) Three-dimensionally rendered images of closed surface area for calculating the SI. (C) Formula of the SI. According to our previous procedure (Sawada et al. 2014), the SI of entire cerebrum and each sulcus was calculated using these formulas from was a modified procedure originally designated by Dubois et al. (2008). cns, coronal sulcus; crs, cruciate sulcus; csss, caudal suprasylvian sulcus; his, hippocampal sulcus; ls, lateral sulcus; olfs, olfactory sulcus; ots, occipitotemporal sulcus; prs, presylvian sulcus; pss, pseudosylvian sulcus; rf, rhinal fissure; rs, rhinal sulcus; rss, retrosplenial sulcus, rsss, rostral suprasylvian sulcus; ss, splenial sulcus.
