# Supplementary material for: Sexual dimorphism of sulcal morphology of the ferret cerebrum revealed by MRI-based sulcal surface morphometry
Source: Front Neuroanat. 2015 May 6;9:55. doi: 10.3389/fnana.2015.00055 (PMC4422084; doi:10.3389/fnana.2015.00055)
Supplement: Supplementary file 3 [file Image3.PDF]

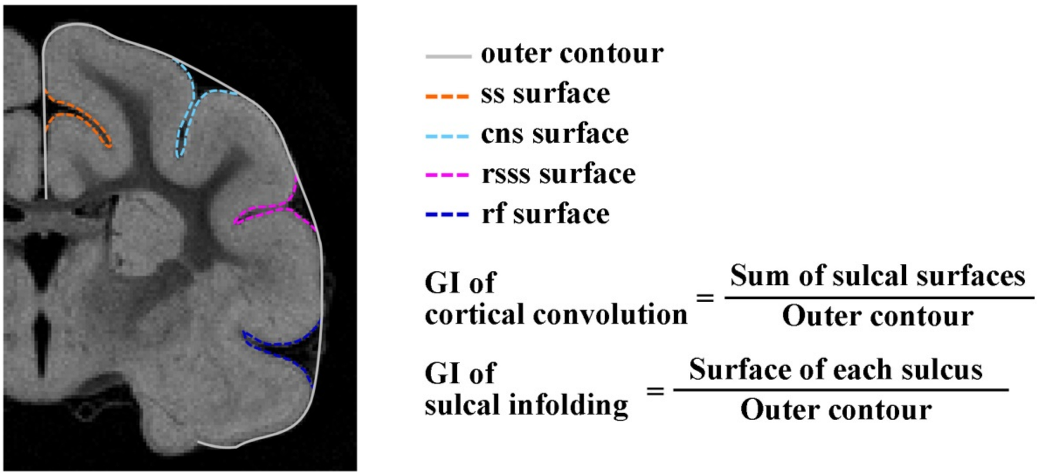

Supplemental Figure 3. Procedures and formulas for calculation of the gyrification index (GI) of the cortical convolution and sulcal infolding frequencies on coronal T_1-weighted (short TR/TE) MRI. The outer contour of the cerebral cortex and each sulcal surface are drawn on a representative coronal MRI. Frequencies of the cortical convolution and infolding of the primary sulci on each MRI images were calculated. cns, coronal sulcus; rf, rhinal fissure; rsss, rostral suprasylvian sulcus; ss, splenial sulcus.
